# From patient advocacy to patient‐driven research: Building active partnerships beginning at the bench to reach the bedside

**DOI:** 10.1002/2211-5463.70318

**Published:** 2026-08-20

**Authors:** Jenica H. Kakadia, Rachel Heilmann, Maximiliano Barrientos, Laura MacNeill, Ingrid Vallee, Sarah K. Schultz, Patrick O'Donoghue, Angelica A. Moresco, Victoria M. Siu, Ilka U. Heinemann

**Affiliations:** ^1^ Department of Biochemistry The University of Western Ontario London Canada; ^2^ The Rory Belle Foundation Denver CO USA; ^3^ Charcot‐Marie‐Tooth Research Foundation Atlanta GA USA; ^4^ Children's Health Research Institute London Canada; ^5^ Department of Chemistry The University of Western Ontario London Canada

**Keywords:** patient advocacy groups, patient‐driven research, patient panels at scientific conferences

## Abstract

Patient advocates offer lived experience that can shape research priorities, methods, and outcomes. Patient engagement contributes valuable resources to research, including meaningful additions to research design, definition of research priorities, and dissemination of outcomes. It is therefore critical that patients and patient advocates be part of the biomedical research process; which can be achieved by changing the perspective of patients as the recipients of research outcomes, to being partners in the process. Patient, clinician, and researcher interactions are particularly important in rare disease research, where little funding is allocated to each disease, making the definition of research priorities even more important. Here, we outline how patients can contribute to biomedical research and provide a step‐by‐step guide for patient engagement at scientific conferences.

## Graphical abstract

Research is strongest when conducted alongside patients, not just about them. Patient research organizations help integrate patient perspectives into research priorities, study design, and scientific meetings, leading to meaningful patient outcomes and development of relevant therapies. We provide practical recommendations for building lasting partnerships between patients, clinicians, and researchers.

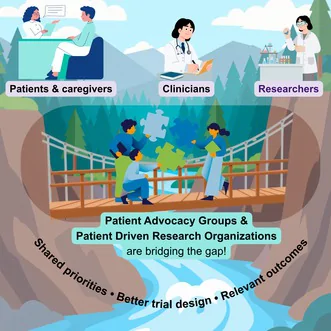

**Abbreviations**
CBPR: Community‐based Participatory Research
CMTRF: Charcot–Marie‐Tooth Research Foundation
FDA: Food and Drug Administration
G‐tubes: gastrointestinal tubes
HIV/AIDS: Human Immunodeficiency Virus/Acquired Immunodeficiency Syndrome
KARS1: lysyl‐tRNA synthetase
NARS1: asparaginyl‐tRNA synthetase
NIH: National Institutes of Health
PAGs: patient advocacy groups
PDROs: Patient‐Driven‐Research Organizations

Biomedical research has traditionally prioritized mechanistic understanding and often emphasizes uncovering molecular pathways and genetic determinants as the foundation for therapeutic discovery. Although this approach advances fundamental knowledge, it also contributes to a persisting gap between research priorities and patients' lived experiences. Mechanistic approaches and readouts of cellular function yield invaluable insights and are necessary for development of therapeutic interventions. Basic research and biomedical studies in the laboratory, however, often do not result in meaningful outcomes to improve the daily lives of patients. Indeed, this issue extends beyond basic research; many clinical trials fall short in the design stage when determining relevant research outcomes [1, 2, 3]. We found that often clinicians, researchers, and patients have different priorities. Researchers want to find the ‘why and how’ of a disease allele, often without considering the impact and relevance of their research on patient lives. Clinicians, and even specialized medical geneticists, on the other hand, see many patients and often do not have the bandwidth and time to research every symptom associated with specific disease and disease‐specific gene or variant. Often, clinicians focus on the symptom they specialize in managing and may not capture the full burden of the collection of symptoms. Patients and patient caregivers are focused on function and quality in day‐to‐day life. Specifically, patients and caregivers prioritize improving the symptoms that impact and interfere with their lives.

Roughly, 300 million individuals worldwide are diagnosed with a rare disease, which impairs function, decreases independence, and contributes to emotional and logistical complexities of navigating a condition with limited resources [4]. The annual economic burden of approximately 300 rare diseases in the United States approached US $1 trillion. These astonishing figures highlight a pervasive crisis [5] and urge a transformation in therapeutic development. Connecting researchers and patients will accelerate discovery by enabling donation of patient‐derived biospecimens to aid in the development of patient‐derived cellular models of disease, organizing patient communities to facilitate enrollment in clinical trials, and by developing awareness in the research community of the most pressing needs of the patient community. Even though patient voices are increasingly heard and incorporated into research, many research communities do not actively seek out patient perspectives—and many clinicians work in isolation from the research community as well. However, patient advocates and patient advocacy groups play an essential role in bridging the gap between researchers and patients. This article aims to highlight existing strengths and weaknesses in patient engagement in scientific research, framed by the authors' lived experience.

## Recognition of patient involvement by regulatory agencies and the scientific community

Although patient‐reported outcomes are increasingly considered in the literature, they remain less central than clinician‐developed or laboratory‐based endpoints, leading to rare symptoms and adverse events being underrepresented, especially in rare‐disease research. Critical insight, such as increasing research emphasis on symptoms affecting patient quality of life, including mobility preservation, pain management, and communication impairments [6], has led to the development of patient‐reported outcome measurements [7].

### Regulatory recognition of patient perspectives

The Food and Drug Administration (FDA) [8], European Medicines Agency [9], and most recently the United Kingdom [10] have issued guidance for industry and academic collaborators on how to obtain and incorporate what matters to patients, technology, and clinical outcome assessments from multiple perspectives [6, 8, 11, 12]. Nonetheless, we find that many researchers are not aware of these guidelines and do not incorporate them into their research proposals. A step in the right direction is the encouragement and acknowledgement of patient engagement and training modules as a part of grant applications that has been adapted by several national funding agencies (e.g., Canadian Institute of Health Research).

### Shifting research expectations and scientific publications

Similarly, scientific journals now frequently encourage (and sometimes require) explicit consideration of patient engagement strategies, signaling a shift from viewing such involvement as optional to essential [13]. More recently, public funding agencies have shown a similar shift in acknowledging and supporting the engagement of patient partners [14]. These considerations acknowledge that scientific rigor and patient relevance are mutually reinforcing, and benefit clinicians, patients, and researchers alike [3].

### Historical recognition of patient advocacy

Regulatory guidance has provided the proverbial green light for patients or patient advocacy groups (PAGs) to co‐exist and drive the therapeutic development from concept to reality since the 1990s [15, 16]. PAGs have existed since the 1950s with the formation of the Cystic Fibrosis Foundation [17] and expanded further in the 1980s with the HIV/AIDS (Human Immunodeficiency Virus/Acquired Immunodeficiency Syndrome) activism movement [18]. Both PAGs were founded as awareness groups but spurred a real effort to include patients in research and ultimately drug development [15]. They now serve as examples as what is possible when rare disorders, or in some cases ignored diseases, become common and visible. Subsequent formation of consortiums coupled with the advent of broader genetic testing and targeted genetic medicines has created an opportunity for PAGs to drive research and therapeutic development forward to the benefit of patients [19]. The path ahead has already been paved with effective integration of PAGs. Together, we already stand on the shoulders of giants with the collective knowledge that this collaborative structure can succeed, not whether it will.

## Bridging the gap between patients and researchers to address challenges in rare disease

Engagement with both patients and their caregivers helps bridge the divide by reframing research priorities around lived experience rather than only considering biological tractability or clinician centered perspectives. The framework for successfully connecting researchers to patient needs is encapsulated by the motto that ‘research should be alongside patients, not just about patients, which encourages incorporating subjective outcomes in assessing therapeutic efficacy alongside measurable parameters. For example, quality of life surveys can be utilized alongside biochemical and biophysical measurements in the assessment of clinical outcomes [20].

### Patient‐reported and observer‐reported outcomes

Validated and standardized patient reported outcome measurements [21] and observer‐reported outcomes (e.g., by patient caregivers, educational partners or clinicians) take lived experience into consideration to help assess overall health status, symptom severity, and psychological function, among other aspects of health‐related quality of life [6, 22]. Developing patient reported outcome measurements, which already can be time and resource intensive, poses additional challenges when considering rare diseases, due to disease heterogeneity and small patient populations [23]. We have noted particular challenges in severe neurological pediatric rare diseases where current validated assessments are not sensitive or specific enough to capture baseline, let alone symptom improvement with therapeutic interventions [12, 23]. In these circumstances, PAGs are uniquely positioned to articulate collective priorities of the population [24, 25]. This approach can inform both the scientific questions and clinical care that target burdensome symptoms.

### Structural and logistical barriers to collaboration

The question on how PAGs, researchers, and clinicians connect and serve each other remains ill‐defined but often relies on grit, determination and a bit of circumstantial luck. The shift to patient centered research is met with persistent structural challenges, as patients and their caregivers may be positioned as passive recipients of research outcomes rather than active contributors to the research process. Many basic science researchers, for example, may not receive formal training on collaborating with clinicians or patient advocacy teams. Seemingly small changes in language, such as referring to disease alleles as alleles or variants, rather than mutants or mutations can make a difference in how results are communicated in a respectful, inclusive, and understandable way. Additionally, translational research timelines [23], which can span many years or decades, may not be able to keep up with the evolving needs of patients navigating progressive disease. Advocacy partners can help inform trial design and feasibility, and offer input on inclusion and exclusion criteria, especially where relevant outcomes may be difficult to determine or where natural history of rare disease progression may not be formally documented yet. Additionally, these groups help highlight important limitations to consider. For example, in NARS1 (asparaginyl‐tRNA synthetase, NARS1 disease) or KARS1 (lysyl‐tRNA synthetase) ultra‐rare neurological disorders, those affected are often children with complex medical needs, including seizure burden, gastrointestinal tubes (G‐tubes) requiring electronic pumps for formula administration, wheelchairs or walking devices, and daily medication administration. This can all put an additional hardship on families who may wish to participate in research but may not be able to due to cost, health risk, or logistical burden. These issues are amplified due to few patients living in proximity of each other for centralization of in‐person studies. For example, the lack of geographically dense populations increases the travel burden for rare disease populations to access clinical trials, expert care, or for research collaborations. Not accounting for these limitations can significantly reduce a study's reach and may lead to false conclusions, as the study may unknowingly exclude entire groups of what can already be a very niche yet heterogeneous disease population [3]. Patient advocacy organizations can collect and present these data to help shape more meaningful research.

## The role of patient advocacy groups in research

There are over 10 000 rare diseases, with an estimated 80% of these having genetic causes, and about 95% of without approved treatments [26]. Funding for rare disease research, particularly when facing knowledge gaps, is sparse, despite dedicated seed funding opportunities. Analysis of the NIH (National Institutes of Health) funding landscape over the past two decades reflects greater funding support for infectious diseases, compared to diseases with Mendelian inheritance, to alleviate perceived burden on public health systems [27].

However, the inability to recognize certain rare diseases with a disproportionately large impact on quality of life for both patients and their caregivers negatively affect informed funding decisions. In addition to understanding patient experiences, advocacy groups can help researchers procure relevant biospecimens and patient‐derived cells, which are essential for developing models of disease to rapidly evaluate potential therapeutic approaches that can then be assessed in animal models and ultimately in clinical trials. Groups such as The Rory Belle Foundation (NARS1, asparaginyl‐tRNA synthetase) and Charcot–Marie–Tooth Research Foundation (CMTRF) also provide critical seed funding in circumstances where funding for rare disease research can be difficult to obtain [25]. The goal of the seed funding is to generate data that can be leveraged to develop larger research grant proposals on rare diseases and increase chances of funding success [28].

Patient advocacy groups can lessen the burden of self‐representation for patients, while also bridging communication gaps with clinicians and researchers. In our experience, researchers are often tense in direct interactions with patients, not out of indifference, but because most receive little to no formal training in patient communication. Unlike clinicians, researchers are rarely taught bedside manner or how to navigate emotionally sensitive conversations, and many are acutely aware of this limitation, despite genuinely wanting to communicate meaningfully with patients and incorporate patient perspectives into their work. Patient advocates can play a critical role in bridging this gap, serving as intermediaries who facilitate understanding, foster trust, and help translate between the cultures of research and patient experience. Meanwhile, clinicians often have limited time and capacity to engage deeply with basic science research due to the demands of patient care and heavy clinical workloads. As a result, they may prefer research findings to be distilled into clear, practical, and clinically relevant information. There can also be frustration with the pace of scientific progress; while researchers may work toward long‐term breakthroughs, clinicians and patients are often focused on interventions that can improve quality of life in the immediate present. Because many patients may never directly benefit from potential future treatments within their lifetime, clinicians and patients may prioritize actionable therapies and supportive care available today. Bridging these differing perspectives requires mutual understanding, effective communication, and individuals who can translate between the long timelines of research and the urgent realities of clinical care.

### Patient expertise informing care infrastructure

**Fig. 1:**
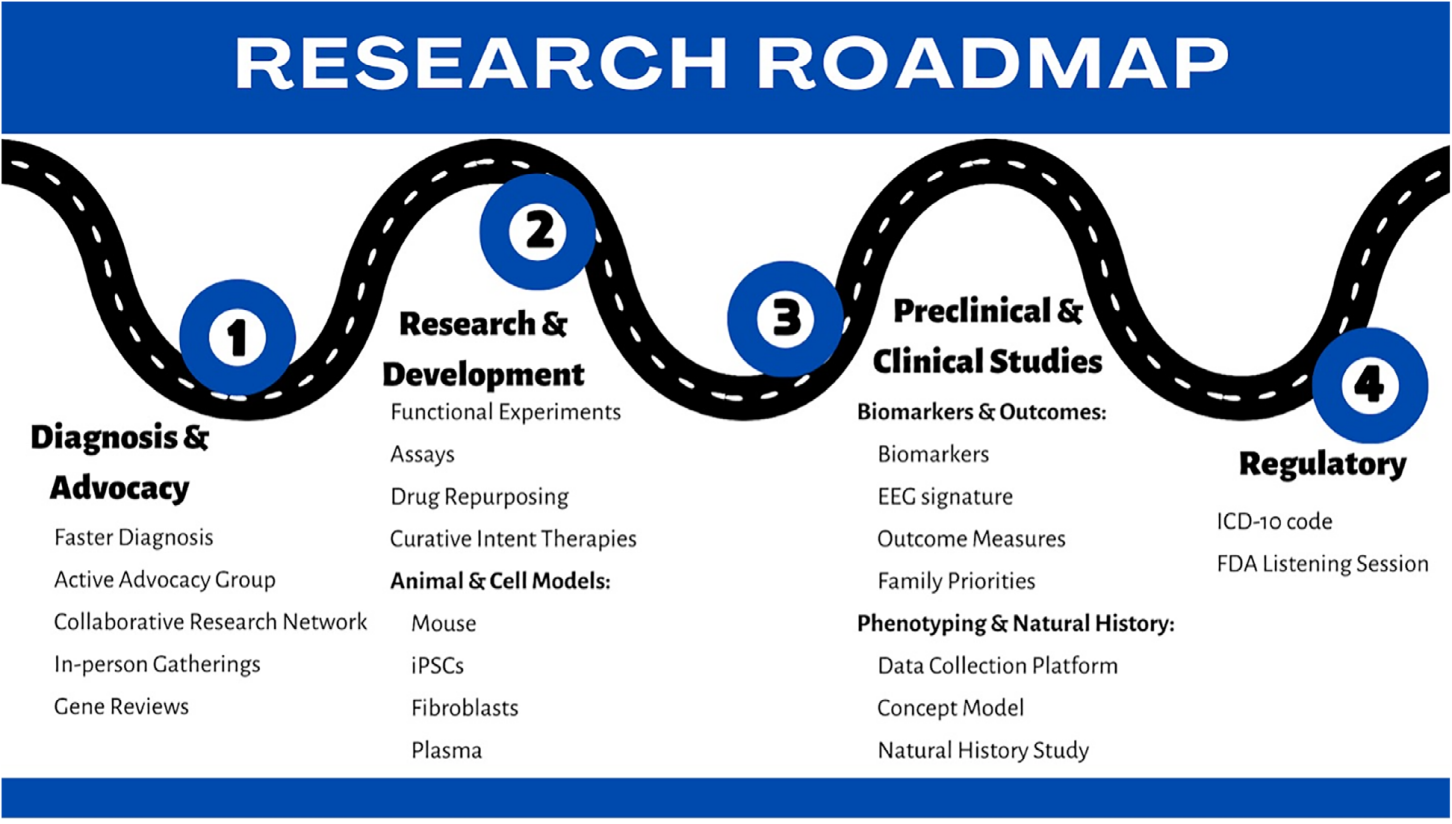
Summary of therapeutic development pathway. Example of a research roadmap for Patient‐Driven Research Organizations.

Typically untrained from a traditional academic scientific perspective, PAG leaders and families have the expertise in the understanding of their own or their family member's genetic condition. They can often provide a more comprehensive viewpoint than an individual specialist physician [19]. Both The Rory Belle Foundation and CMTRF have built communities through maintaining an online presence, building capital, and centralizing critical preclinical, and clinical trial resources. They have laid the strategy for therapeutic development, collected patient and caregiver data through registries, and established biobanks for DNA, live cells, and postmortem tissues [25, 29, 30, 31, 32] (Table 1). These have been previously established as a blueprint for success. However, each must determine what fits their specific goals and leadership in framing those cross‐collaborative relationships. For families affected by diseases without treatments, there are no pretenses. The priority is alleviation of the symptoms and for a cure for the disease that plague them or their loved ones. Expectations and priorities for engagement in transparent conversations with researchers and clinicians are necessary to achieve the shared goal of rapid therapeutic development to reduce, ameliorate, or cure disease. With this, they are driving research, transitioning to become Patient‐Driven‐Research Organizations (PDROs), using the trained expertise and newly formed relationships to create a research road map, complete with the preclinical tools necessary to execute when clinical trials become available (Fig. 1) [29, 30, 31]. For The Rory Belle Foundation, this has meant being present at scientific conferences to search for future partners. For more established PDROs, such as the CMT Research Foundation, partnerships have been long‐standing.

**Table 1:** Potential contributions of PAGs to clinical trial readiness. Displayed information is a summary of potential opportunities identified from the literature.

Example	Contribution	Impact on clinical readiness
Data Infrastructure	Patient Registries & Natural History Studies	Collects essential longitudinal data on disease progression, which helps regulators (like the FDA) understand what a “successful” treatment looks like.
Research Funding	Seed Grants & Translational Research	Provides early‐stage funding for “proof‐of‐concept” studies that are often too risky for traditional venture capital or large pharma.
Trial Design	Patient‐Centric Outcome Measures	Ensures that clinical trials measure what matters to patients (e.g., quality of life, mobility) rather than just laboratory biomarkers.
Recruitment	Trial Awareness & Enrollment	Acts as a trusted communication channel to identify and recruit eligible participants quickly, significantly reducing the time and cost of trials.
Biobanking	Collection of Biospecimens	Facilitates the collection of blood, tissue, or genetic samples, providing researchers with the raw materials needed for drug discovery.
Regulatory Input	U.S. Food and Drug Administration (FDA) and European Medicines Agency (EMA) engagement	Provides the “patient voice” during regulatory meetings, helping agencies weigh the benefits of a new drug against the risks of leaving a disease untreated.
Network Building	Collaborative Research Networks	Connects isolated academic researchers with industry partners and clinicians to create a ‘bench‐to‐bedside’ pipeline.

### Mutual benefits of research partnerships

What is to be gained by such disruption in the status quo is a symbiotic, enduring relationship connecting patients, researchers, and clinicians. By creating a centralized platform, the intent is to provide the space (virtually and in‐person) for researchers and clinicians to share ideas, expertise, results, and future directions. Researchers gain access to critical biospecimens and de‐identified patient data–all collected and owned in partnership between the PDRO leaders, and the community affected by the disorder. In return, researchers can clarify research questions that align work with real‐life meaning for patients, to publish those results, and to turn seed funding into larger grants with the aim of translating discoveries at the bench to therapies at the bedside. Clinicians have the opportunity to provide insight to larger patient populations of similar conditions and symptoms and gain specialty knowledge about rare diseases to publish or develop clearer care guidelines or *Gene Reviews* that allow providers to support families following a diagnosis (for example a NARS1 gene review [33]). Finally, the patient community contributes to knowledge about their rare condition through direct interactions with researchers and clinicians. All parties have the chance to benefit if they are open to the experience.

Often, these relationships can lead to something greater. Copublications with patients or PDRO leaders, physicians, and basic researchers highlight the shared investments in each other. Publications may focus on exactly what regulatory agencies are requesting, the patient voice. For example, integrating patient caregiver interviews into published literature can lead to a more complete view of disease symptoms and can fill crucial gaps in disease state knowledge [34, 35]; qualitative interviews of patients and patient caregivers can be conducted to understand the breadth and depth of symptoms and the impact on the individual and families. Clinicians may also partner with PDROs to publish natural history studies or to highlight how the perspective of symptom burden is different [36, 37]. All this information can in turn facilitate more effective experimental and clinical trial design by identifying the time course of symptom onset, and tissues most affected by disease, potentially helping to pinpoint the best modes and timing for therapeutic delivery.

## Patient involvement in scientific conferences

Historically, patient‐centered conferences have played a great role in connecting patients with each other and with clinicians. Similarly, researchers meet at scientific conferences to exchange ideas and build collaborations, but patients and researchers rarely interact. In recent years, an effort has been made to include patient voices at scientific conferences, spurring new partnerships between patients, researchers, and clinicians. These meetings expanded awareness of rare disorders through partnership with disorder‐specific community members participating in patient panels at clinical and scientific‐based conferences. This provides a broader audience where those affected have the opportunity to share their journey with rare disease and raise awareness for research funding programs or preclinical tools PDROs are developing.

It should be noted that moderating and creating a patient panel may be different than a typical scientific panel for conferences. Contributing families are usually without scientific background and have had different experiences in their rare disease journeys. It is important to engage in thoughtful questions that support them, as well as help them connect with the audience. For many patients and patient caregivers, a patient panel may be the first time their personal journey is shared to a broad audience. It may also be the first place a researcher hears of the hardship of caring for or the death of a loved one from the disorder they are studying at the bench. Patient panels are meant to spotlight the reality of the lives effected to those who do not live it and to inspire the direction of science. Thus, there are several key considerations to review as research works to build bridges with patients and the broader patient‐community. Based on our own experiences and to help inspire and encourage more engagement between researchers and patients, we developed a step‐by step guideline on how to incorporate patient panels into a scientific research conference (Data S1). Our guide outlines detailed procedures for patient recruitment, addressing the financial obligation of the scientific community to cover costs for patients, family members, and caregivers in addition to ethical considerations. Particularly, patient engagement must be approached with respect and intentionality by centering patient dignity and setting clear goals when integrating it into the overall program. Patients and patient advocates should be offered appropriate compensation to ensure fair and accessible participation. Further, it is important to establish a well‐structured organizing team by considering individual roles in local logistics, patient liaison, and effective moderation. Recruitment for the panel should prioritize diversity in representation by involving patient advocacy groups and clinicians, and supporting panelists with clear communication, consent, and accessibility accommodations. When deciding on panel topics, it is imperative that participants are given opportunities to review and shape the discussion in advance. The panel itself should be conducted in a well‐moderated environment that centers patient voices and allows space for emotional expression. The most meaningful panels facilitate thoughtful and inclusive dialog with the audience.

Following the panel, gathering feedback helps create opportunities for continued engagement while documenting key outcomes and lessons learned. In the guide, we additionally list common pitfalls, such as inadequate planning, insufficient financial resources, poor communication with panelists, and treating panels as nonessential, to be actively anticipated and avoided. If considering a virtual or hybrid panel, additional considerations such as technical readiness of the moderators and managing fatigue in different time zones should be made. All aspects of planning and executing patient panels should be done with ethical considerations, including informed consent, respect for autonomy, and protection of privacy. Feedback from our recent patient panel, developed using the concepts presented in the guide, at the International Symposium on Aminoacyl‐tRNA Synthetase (AARS2023, Grand Bend, Ontario, Canada) described the patient panels as ‘a highlight in the program’ [38], demonstrating the impact collaborations between patients and researchers can have on both groups.

## Barriers to patient involvement in research

Based on our perspective and experiences, we have found that connecting patients with researchers serves to propel both drug discovery and basic biomedical studies to understand the root cause of genetic diseases. While our guide may help to increase interactions between researchers and patients, several challenges and barriers should be recognized, so that these can be acknowledged or overcome to better integrate patient perspectives in research with the goal of providing therapeutics to improve patient lives and quality of life. Additionally, just as patient panels can have meaningful benefit so can integrating researchers directly into the community. Using PDRO‐led virtual or in person researcher and family conferences is a key element in improving an understanding of the research and collaborative efforts for those directly affected. These opportunities provide a platform for a broad patient and patient caregiver audience to begin to grasp complex topics and ask questions related to the disorder they face every day.

### Barriers in communication

Barriers in communication between researchers, clinicians, and the general public audience can be difficult to bridge. Health literacy, a determinant of health [39], can be supported by organizational training processes to encourage informed decision‐making and disseminating information in a culturally and linguistically informed manner. Most PDROs are run by volunteer‐family members without a background in science and health care. Additionally, many rare disease communities are multilingual, necessitating translators for communication with each other and with researchers who may not speak the same language. This must be considered, in addition to ensuring conversations are understandable to a lay audience. Guidance for communicating science effectively includes accounting for audience values and prior beliefs, using relatable language, and framing information in ways that connect to real‐world decisions [40]. Barriers, including scientific jargon, can affect how rare disease community members participate in research opportunities or their willingness to fundraise for key preclinical research studies and resources [41]. Indeed, scientific principles and language contributes to documented reductions in comprehension and engagement in low health literacy populations [42]. To overcome this barrier, several resources are available including the Cochrane Collaboration's in‐depth guideline for writing plain language summaries [43].

Scientific and clinical advisors can often help translate complex topics to remedy communication barriers. This is key when engaging in Community‐based Participatory Research (CBPR), a partnership‐based approach to research involving community members, organizational representatives, and researchers as equal partners [44]. Another limitation is the time needed to reduce this barrier. A consideration for improvement in cross‐communication processes can be leveraging new artificial intelligence tools to generate lay language summaries, with studies showing high accuracy, relevance, transparency, and accessibility using these emerging tools [45]. Support from interns or students working with PDROs can also alleviate the unseen workload of PDRO volunteer members. While both support systems still require oversight and editing before publication of material, it can reduce the load on researchers, clinicians, or PDRO leaders.

### Academic conflicts in publication

Another constraint of collaboration between academic researchers, clinicians, pharmaceutical companies, and patients is the disagreements over intellectual property or conflicts of interest that may arise between parties. Broad transparent sharing of prepublication data is not the usual process in academia as career and ultimately financial incentives are tied to publications. The well‐known adage ‘publish‐or‐perish’ to propel a researcher's career limits educational academic activities and can, at times, make it difficult to have open communication between PDROs and researchers/clinicians with so much at stake. It is important in these cases for PDROs to have bylaws, nondisclosure agreements, or other agreed upon guidance that allows for open dialog, participation, and sharing expertise. Another area of conflict may be between PDRO board and community members in choosing who to fund. Often for rare diseases, the volume of experts is narrow, and those are often part of the Scientific and Medical Advisory Board for PDROs. Conflict of interest mitigation techniques must be implemented within the advisory board through policies around reviewing grant applications or signing conflict of interest statements in order to keep objectivity in place [46]. Fostering effective collaboration between different groups—particularly when they are not yet familiar with one another—should be established early to build strong, long‐term relationships of trust. Data sharing, including negative or neutral results and troubleshooting insights, should be encouraged between researchers and PDROs. PAGs cannot afford to wait for each group to independently troubleshoot the same assays. By promoting collaborative approaches, PDROs can help accelerate research progress and the generation of meaningful results.

### Conflicting perspectives among communities

Finally, distinct and personal disagreements can and often do occur directly within the rare disease community. Rare diseases are complex with a broad heterogeneous symptom impact as well as unique cultural impacts in some cases. Divergent priorities among patients, caregivers, and researchers are well‐described [47]. Some patients and patient advocates may prioritize direct improvements in day‐to‐day life, while others may want to invest in a long‐term cure (even if it may not be attainable within their lifetime). For those within PDRO leadership, making decisions on how to invest monetary resources may be one of the most difficult challenges. In such situations, it is prudent to listen to concerns while ultimately relying on the research road map to build the resources that can lead to the largest gain in advancing therapeutic development [48, 49].

## Conclusion: From advocacy to patient research organizations

The concept of PAGs, not only being advocates, but being active participants in translational research is not a novel concept, but it is game‐changing. PAGs are no longer bystanders, and many have transitioned to become Patient Driven Research Organizations. With structured regulatory guidance from around the world supporting the integration of the patient voice, new therapeutic modalities, and gene‐based diagnosis, the question should not be *‘why are researchers not engaging with patients?’* but rather *‘how are researchers engaging with patients?’* to orient research to impact patient lives and to bring therapeutic opportunities directly to the bedside.

## Conflict of interest

The authors declare no conflict of interest.

## Author contributions

RH, IV, LM, and MB are patient advocates and are associated with rare disease associations; JHK, SKS, POD, and IUH are basic scientists with research programs in rare diseases; and VMS and AAM are physician researchers. JK and RH drafted the article; VMS, AAM, LM, and IUH edited the article and drafted the supplement; MB, IV, SKS, and PO edited the article.

## Supporting information


**Data S1.** Patient panel guide.

## Data Availability

Data sharing is not applicable to this article as no new data were created or analyzed in this study.
