# Supplementary material for: From patient advocacy to patient‐driven research: Building active partnerships beginning at the bench to reach the bedside
Source: FEBS Open Bio. 2026 Aug 20:10.1002/2211-5463.70318. Online ahead of print. doi: 10.1002/2211-5463.70318 (PMC13492564; doi:10.1002/2211-5463.70318)
Supplement: Supplementary file 1 [file FEB4-9999-0-s001.docx]

**Supplementary Information for:**

**From patient advocacy to patient driven research:**

**Building active partnerships beginning at the bench to reach the bedside**

Jenica H. Kakadia^1*^, Rachel Heilmann^2*^, Maximiliano Barrientos^3^, Laura MacNeill^3^, Ingrid Vallee^2^, Sarah K. Schultz^1,4^, Patrick O’Donoghue^1,5^ Angelica A. Moresco^4^, Victoria M. Siu^1,4#^, and Ilka U. Heinemann^1,4#^

^1^ Department of Biochemistry, The University of Western Ontario, London, Ontario, N6A 5C1, Canada

^2^ The Rory Belle Foundation

^3^ Charcot-Marie-Tooth Research Foundation

^4^ Children's Health Research Institute, London, ON, Canada

^5^ Department of Chemistry, The University of Western Ontario, London, Ontario, N6A 5C1, Canada

* These authors contributed equally.

\# To whom correspondence should be addressed: V.M. Siu [vmsiu@uwo.ca](mailto:vmsiu@uwo.ca); I.U. Heinemann [ilka.heinemann@uwo.ca](mailto:ilka.heinemann@uwo.ca)

**Supplementary Patient Panel Guide: how to incorporate patient voices into scientific conferences**

1. **Introduction: Purpose of Patient Panels**

Patient panels bring the patient voice directly into scientific conferences, helping researchers understand the real-world impact of their work and guiding research priorities based on patient needs, experiences, and testimonials. For many researchers these sessions are the first time they meet patients or family members affected by the condition they study, which can fundamentally shift or re-prioritize how scientists think about project timelines, communication and dissemination of their research findings, and their work more broadly, especially regarding the impact of their research on patient lives.

Patient panels also help build trust and relationships between the research and patient communities, highlight outcomes that matter most to patients, and ensure that research agendas are grounded in lived experience rather than solely in scientific curiosity. Patient panels often include patients themselves, and patient advocacy groups, family members, and patient caregivers.

These guidelines are intended to support conference organizers, research, and patient advocates in planning patient panels that are respectful, meaningful, and logistically sound. We believe that these guidelines, based on our experience in organizing patient panels, can be replicated across national and international scientific meetings to ensure establishment of new and growing bonds between patient communities struggling with disease and researchers that study the disease and therapeutic interventions as well as cellular and molecular components that underlie disease progression and pathology.

2. **Before you start**

Patients and patient advocates need to be treated with the utmost dignity and respect. They commit their time to help scientists, not the other way around. As researchers we should be asking ourselves how we can help patients, not how they can help us. Always be aware that they may not see a cure or treatment in their lifetime, and honor their bravery of their willingness to commit precious time, engagement, and resources to helping further research towards a cure.

Before committing to a patient panel, organizers should have:

- Clear goals for what they hope participants and the audience will gain (e.g., understanding daily life with the disease, research gaps and priorities, current standard of care, barriers to care or new therapeutic development).
- Organizational commitment to provide appropriate financial support, accessibility accommodations, and follow-up include gathering feedback and to express gratitude and encourage continued participation in connecting with researchers.
- Agreement from conference leadership that the panel is a core part of the program, not a token or last-minute add-on.

3. **Financial resources**

Travel, accommodation, conference registration for the day and food for each patient, family member, care giver, and/or patient advocate must be fully covered. There should be a token compensation for the time, as many patients and patient advocates take time off work for these events. Compensation amounts may vary by country and institutional policies. When possible, consult with patient advocacy organizations about appropriate rates and preferred forms of compensation (e.g., honoraria, stipends, gift cards, per diems).

Budgeting and funding sources

- Conference budgets: The budget should include specific and dedicated line items for patient engagement or community participation.
- Potential Funding sources:
  - National health research agencies (e.g., dissemination/engagement supplements or conference grants).
  - Disease-specific and umbrella patient organizations or foundations that support patient involvement in research.
  - Academic journals often contribute funding to support scientific conferences, and they may be willing to provide additional funds to support a patient-panel.
  - Pharmaceutical/biotech sponsors, industry partners, and private donors are all important potential funding sources, however, arrangements must be made with clear boundaries to preserve independence and avoid conflicts of interest.

4. **Organizers and timelines**

One organizer should be local to the conference; and direct the organization of accommodation and travel. Ideally, the organising team includes

- A local logistics lead (travel, accommodation, on-site support)
- A patient/community liaison with experience working directly with patients and families (e.g., someone from a patient advocacy organization).
- A scientific lead who can help integrate the panel into the scientific program and communicate with session chairs.
- A moderator (this may be a researcher, clinician, patient liaison or another experienced facilitator).

Recommended planning timeline:

- 6-12 months before the conference: Confirm leadership buy-in, identify funding, and secure session slot.
- 4-6 months before: Begin recruitment of panelists and moderator.
- 2-3 months before: Confirm travel and accommodation, draft questions for panel discussion, and share expectations with panelists.
- 1-2 weeks before: Final logistics email, test any virtual platforms (if hybrid/virtual), and hold a pre-panel call to ensure all patient needs or potential concerns are addressed and to review the plan and schedule for the patient panel at the conference.

5. **Recruiting patients and patient advocates**

- Aim for a panel of 5 or more members.
- Aim for local members, if possible, to lessen the burden of travel and time commitment. Of course, where feasible, including members from the broader international patient community can help patients and researcher understand the global reach of the disease.
- Patient advocacy organizations will often be interested to participate and can help identify local patients, clinicians, and caregivers that may wish to participate.
- It is helpful to have one physician (or physician scientist) on the panel, as they can add medical and patient care perspectives. Physicians have been trained to work with patients and are often more appropriate in their interactions than bench scientists.
- Reach out to physicians and physician scientists, who may know of patients active or interested in research.
- The initial recruitment should be done through patient advocacy organizations or physicians/physician scientists, or by using relationships that are already existing (e.g., based on your previous interactions with a patient community).

Additional considerations when recruiting panel members:

- Strive for diversity across age, gender, race/ethnicity, socioeconomic background, and geography where possible.
- Again, where possible, include a mix of people at different disease stages (newly diagnosed, living long-term with the condition, caregivers of children or adults, family members, etc.)
- Consider whether both patients and caregivers should be represented, and if so, how to balance voices.
- Be transparent about what participation involves (time commitment, public nature of the session, recording, photography, and dissemination in publications or social media).

***Important notes***:

- Be very respectful of relationships that have already been established. For example, some patients or patient advocates may want to mostly interact with “their” contact person, such as the physician or physician scientist they have been working with. Ask what they prefer.
- Patient advocacy organizations often have much more experience in patient panels and speaking to the public than most scientists have when interacting with patients. Let them take the lead if they wish to do so.
- Refer to disease causing alleles as “variants”, *not* mutations or mutants.
- Ask panelists how they prefer to be identified (e.g. “person living with “disease X”, “Disease X patient”, “parent of a child with disease X”) and use their preferred language consistently.
- Accessibility must be considered as an essential part of the organization process. Many rare diseases are associated with disabilities, some of which may be invisible.

6. **Preparing for the panel**

- Some patient advocates do not have a scientific background and do not necessarily know everything about the science underlying the disease (i.e., mechanism, genetic, or cellular components linked to disease). To facilitate collaborations, include a clinician or clinician-scientist contact to the conference handbook for researchers to communicate with if they have specific science questions.
- If you want to record the panel or take pictures, get consent from each panel member.
- Once you have established a panel, contact members to introduce them to each other if this is desired, and offer to share contact information with each other (with consent).
- For the introduction at the conference, it is helpful to have a brief biography and a picture for each panel member. You can post the pictures and brief biographies on the projector screen during introductions. Send an example bio to help guide patients and patient advocates in preparing the information.
- Send out detailed information on the agenda the day of the panel, including time and location of the patient panel and availability of coffee breaks and lunch or dinner as appropriate.
- Send out tentative questions that will be asked at the panel and ask for feedback on the inclusion/ exclusion on questions and topics.

Additional preparation steps:

- Ask about and plan for accessibility needs (mobility aids, wheelchair access, sensory sensitivities, dietary restrictions, need for breaks, interpreter services, etc.).
- Share a brief overview of the conference (audience type, scientific level, other relevant sessions) so panelists know what to expect at the meeting.
- Offer an optional pre-panel call or virtual meeting so panelists and the moderator can meet, review questions, and build rapport.
- Clarify whether any media or press will be present, and what that means for panelists’ comfort and consent.
- Plan the session length (often 60–90 minutes) with coffee breaks if needed, recognizing that sharing emotional experiences can be tiring and important connections and relationships can be made during nourishment breaks.
- When possible, offer to cover conference travel and accommodation costs upfront, as some patient advocates may otherwise need to pay these expenses out of pocket in order to attend.

Sample pre-panel email topics:

- Session date, time, and location.
- Format: Panel member introductions (10 min), panel discussion (20 min), moderated Q&A (20 min), audience questions (15 min).
- Prepare tentative questions in advance and invitation for panelists to suggest edits or additional topics.
- Logistics: travel, accommodation, per diem/meal information, accessibility details, and contact information for onsite support.

7. **Running the panel**

- Introduce the participants
- If one of the participants has participated in patient panels before, check beforehand with them to ask if they would be willing to be the one who starts out to break the ice.
- Ask the agreed upon questions but let the panel take the lead on who wants to answer what questions – not everyone has to answer every question. Provide ample time and space for everyone to contribute to the discussion, if they wish.
- Allow ample time for patient advocates to share at their own pace. Remember that they are opening up about deeply personal, often painful experiences with a room full of strangers. Be patient with pauses, offer supportive acknowledgment, and never rush their responses. ***Create space for emotion and reflection*.**
- Encourage the audience to ask questions: Well in advance and again at the beginning of the patient panel, remind scientists who want to ask questions to be careful about the way they talk about diseases and variants, something "exciting" for a scientist may be traumatic for a patient or patient caregiver.

Moderator best practices:

- Open by setting expectations for respect, confidentiality (as appropriate), and sensitive language.
- Let panelists know they can decline to answer any question, and that it is fine to take a moment if they become emotional.
- Balance speaking time so that all panelists who want to share have the opportunity, gently redirecting if one person or the audience dominates. Enlist a session chair who can help with this in the event a question from the audience goes overly long.
- Have 2–3 backup questions ready in case audience questions are slow to begin or go off-topic.
- If a question is phrased insensitively or is too technical, the moderator can reframe it in more appropriate language before inviting panelists to respond.

Room and setup considerations:

- Ensure the stage and seating are accessible, with ramps and space for mobility devices.
- Provide water, tissues, and, if possible, a nearby quiet room where panelists can decompress after the session.
- Check microphones, slides, and recording equipment in advance to avoid technical delays.

8. **Follow-up after the panel**

- Send a Thank you note. If you have pictures of the panel, send them along as well.
- Make sure all travel expenses are reimbursed.
- Ask what improvements they would suggest for future panels.
- Ask if they have any further thoughts they would like you to relay to the research community.

Additional follow-up ideas:

- Share any high-level feedback from conference attendees (aggregated, not individual), so panelists can see the impact of their contributions.
- Offer options for continued involvement (e.g. advisory role, future panels, review of lay summaries or patient-facing materials), without creating pressure to say yes.
- Document key themes or messages that emerged and, with panelist consent, share them with relevant working groups, funders, or research teams.

**9. Common pitfalls to avoid**

Some frequent challenges and how to avoid them:

- Underestimating the time and funding required to properly support participants.
- Treating the panel as a “nice-to-have” add-on rather than an integral component of the scientific program.
- Insufficient moderator preparation or lack of clarity about goals and key messages.
- Not planning for emotional content (no tissues, no quiet space afterward, no check-in with panelists).
- Last-minute logistical changes (room shifts, time changes) that are not communicated clearly to panelists.

**10. Virtual or hybrid patient panels**

When panels are fully virtual or hybrid:

- Test the platform (audio, video, screen-sharing) with panelists in advance and provide simple instructions.
- Offer phone dial-in as a backup if internet connections are unstable.
- Consider fatigue and scheduling: shorter sessions or breaks may be needed, especially across multiple time zones.
- Use features like chat or moderated Q&A for questions, with the moderator curating questions to avoid insensitive or repetitive ones.
- Clarify if the session will be recorded and how the recording will be used and obtain explicit consent.

**11. Measuring impact**

Where possible, consider simple ways to understand and document the impact of the patient panel:

- Include 1–3 short questions in the conference evaluation form (e.g.., “Did the patient panel change how you think about your research?”).
- Collect qualitative comments or reflections from attendees (e.g. “What did you learn from the patient panel?”).
- Track any follow-on collaborations, research questions, or initiatives that emerged from the panel.

These data can strengthen future funding applications and help justify continued or expanded patient involvement in scientific meetings.

**12. Ethical considerations**

Ethical principles should guide all aspects of planning and running patient panels:

- Ensure informed consent for participation, recording, and photography.
- Make it clear that panelists can withdraw at any time without penalty.
- Avoid pressuring panelists to share more than they are comfortable sharing.
- Be mindful of privacy, particularly when discussing children or rare conditions where individuals may be identifiable.
